# A Self-Gelling Powder Based on Polyacrylic Acid/Polyethyleneimine/Polyethylene Glycol for High-Performance Hemostasis and Antibacterial Activity

**DOI:** 10.3390/polym16243516

**Published:** 2024-12-18

**Authors:** Jia Li, Shu Li, Aozhen Zhong, Jun Xing, Ling Li, Cai Wang, Min Zheng

**Affiliations:** School of Biomedical Engineering and Imaging, Xianning Medical College, Hubei University of Science and Technology, Xianning 437100, China; 17305477685@163.com (J.L.); 19507154357@163.com (S.L.); 15264740011@163.com (A.Z.); xingjundsc@126.com (J.X.); liling02@hbust.cn (L.L.)

**Keywords:** self-gelling powder, hydrogel, hemostasis, antibacterial

## Abstract

Powder-based hemostatic materials have offered unprecedented opportunities for the effective sealing and repair of irregularly shaped wounds and high-pressure, noncompressible arterial bleeding wounds caused by surgeries, traffic accidents, and wartime injuries. However, inadequate adhesion to bleeding wounds and poor hemostasis in biological tissues remains challenging. Herein, we report a self-gelling hemostatic powder based on polyacrylic acid/polyethyleneimine/polyethylene glycol (named PPG) for rapid hemostasis and effective antibacterial ability. When deposited on bleeding wounds, PPG powder can absorb interfacial liquid and rapidly swell into a physically cross-linked hydrogel in situ within 2 s to form a pressure-resistant physical barrier. Furthermore, the in vivo and in vitro results indicate that, as an effective sealant, the PPG powder possesses ease of use, excellent hemocompatibility, strong antibacterial abilities, and superior blood clotting abilities. The effective hemostatic sealing capability of the PPG powder is demonstrated in a variety of injury models in rats and rabbits. All of these factors show that, with its superior wound treatment abilities, PPG powder is a profound biomaterial for surgical applications.

## 1. Introduction

Uncontrolled bleeding may lead to a series of severe morbidities, including hypotension, organ failure, and even death from shock [1,2]. Rapid and effective hemostatic intervention is substantial in saving the lives of patients with excessive bleeding before professional treatment [3]. Currently, surgical sutures and staples are often used as the “gold standard” in clinical practice to close wounds and stop bleeding [4]. Although surgical suture treatment has enhanced wound and surgical care to a certain extent, its application in numerous cases entails considerable complications [5]. For instance, preoperative preparation, strict surgical requirements, and long suturing times all make it possible only in the operating room; therefore, it cannot meet the requirements of first aid for the wounded in an emergency [6]. Additionally, surgical suturing is a complex and time-consuming procedure that demands high surgical proficiency [5,7]. Sutures and staples need to be removed 7–10 days after the surgery to prevent infection by foreign bodies, which not only causes secondary pain to the patient but also prolongs the wound healing time [8,9]. Currently, there are many commercially available hemostatic materials, such as gauze, sponges, hydrogels, and powders [10,11]. Gauze and sponges are used to control bleeding through mechanical pressure and by promoting blood cell coagulation [12]. However, the lack of function in adapting to different wound shapes and the adhesion of biological tissues requires external extrusion to fix the material to the damaged tissue, which is not suitable for irregularly shaped wounds and incompressible wounds in vivo [13,14]. Hydrogels are three-dimensional networks of polymers prepared through physical or chemical cross-linking [15]. They can be used as tissue sealants and adhesives to form a physical hemostatic barrier at the bleeding site to control bleeding [16,17]. However, blood flowing persistently from the damaged site can weaken the adhesion between the hydrogel and the wound, resulting in compromised hemostatic abilities [18,19]. Hemostatic powders, such as Quik Clot^®^ and Celox^®^, can be applied directly to fill irregular wounds of various shapes and absorb the liquid component of the blood to effectively control bleeding [20,21]. However, powder-based hemostatic materials fail to maintain a moist environment and lack the mechanical strength to form a physical barrier in bleeding tissue [10]. In addition, some powders with safety concerns are often used, such as those with exothermic characteristics and potential metabolic toxicity, and they may even cause the formation of blood clots [22]. Therefore, it is essential to develop hemostatic powders with rapid hemostasis and good biosafety.

Recent studies have demonstrated that self-gelling powder can rapidly absorb interfacial fluid to form a hydrogel in situ due to physically cross-linked interactions among self-gelling powders, such as hydrogen bonding and electrostatic interaction, and quickly seal damaged tissue despite tissue surface irregularities, giving it potential applications in effective hemostasis [23,24]. For example, a self-gelling hemostatic powder developed by Tan et al. [12] consisted of polyacrylic acid/polyacrylamide/quaternate chitosan (PAA/PAM/QCS). When the self-gelling hemostatic powders came in contact with water, the PAA/PAM/QCS fused and rapidly formed a stable hydrogel in a short time and could rapidly adsorb lots of blood, aggregate blood cells, and platelets. Peng et al. [1] prepared a self-gelling hemostatic powder based on polyethyleneimine (PEI), polyacrylic acid (PAA), and quaternate chitosan (QCS), and it could accelerate hemostasis by absorbing a large amount of blood to concentrate coagulation factors.

Herein, self-gelling PAA/PEI/PEG (PPG) powder with different PEG contents were designed and developed by freeze-drying and grinding a mixture of PAA, PEI, and PEG. Subsequently, the physicochemical and biological properties of self-gelling PPG powders were systematically investigated, including the water absorption capacity, tissue adhesion, and hemocompatibility. Based on these results, self-gelling PPG4 powder was preferred. We further measured the antibacterial ability of the self-gelling PPG4 powder in vitro by using the inhibition rate against both Gram-positive (Staphylococcus aureus) and Gram-negative bacteria (Escherichia coli), and the hemostatic effects were investigated in vivo using four bleeding models in mice and rabbits (the rat liver bleeding model, rat tail bleeding model, and rabbit ear vein and artery bleeding models). The results of the antibacterial performance showed that, compared with the control group, the self-gelling PPG4 powder groups exhibited an excellent bacteriostasis rate against *S. aureus* (97.0% ± 1.7%) and *E. coli* (97.6% ± 1.5%). The in vivo hemostasis test results exhibited that the self-gelling PPG4 powders can show effective hemostasis, especially for non-compressible wounds, unknown bleeding points, and irregularly shaped wounds. Thus, these powders hold promise for wide applications as potential biomaterials for treating acute tissue bleeding.

## 2. Materials and Methods

### 2.1. Materials

Polyethyleneimine (PEI, Mw ≈ 70,000) in an aqueous solution (50 wt%) was purchased from Shanghai Aladdin Biochemical Technology Co., Ltd. (Shanghai, China), and polyacrylic acid (PAA, Mw ≈ 5000) in an aqueous solution (50 wt%) was purchased from Shanghai Macklin Biochemical Technology Co., Ltd. (Shanghai, China). Polyethylene glycol (PEG, Mw = 1000) was purchased from Shanghai Yuanye Bio-Technology Co., Ltd. (Shanghai, China). Gauze was purchased from Nanchang Zhenfeng Medical Equipment Co., Ltd. (Nanchang, China). Escherichia coli CMCC44102 (*E. coli*, Gram-negative) and Staphylococcus aureus CMCC26003 (*S. aureus*, Gram-positive) were provided by Qingdao Haibo Bioengineering Co., Ltd. (Qingdao, China)

### 2.2. Preparation of Self-Gelling PPG Powder

**Figure 1:**
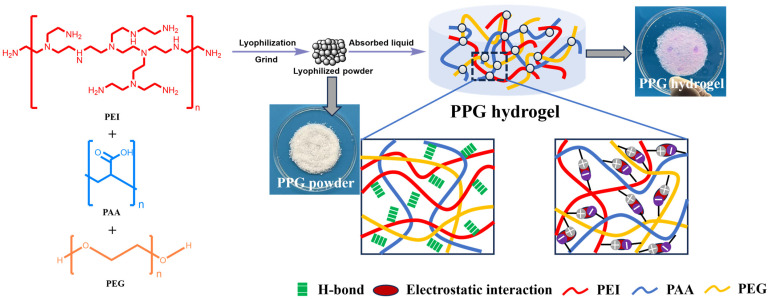
Preparation of the self-gelling PPG powder and self-gelling PPG powder-derived hydrogel.

Briefly, 10 g of PEG was dissolved in 90 g of deionized (DI) water to form a mass fraction of 10 wt%. Then, 50 wt% aqueous solutions of PEI and PAA were diluted to 10 wt% with DI water. Subsequently, 10 wt% PEI, 10 wt% PAA, and 10 wt% PEG precursor solutions were evenly mixed in different volume ratios; the detailed compositions of the self-gelling PPG powders are shown in Table 1. Next, the obtained mixtures were immediately immersed in liquid nitrogen for about 20 min and then transferred to a vacuum freeze dryer and freeze-dried for 72 h. Finally, the obtained freeze-dried samples were ground with a mortar to obtain PPG powders (Figure 1). The self-gelling PPG powders were named PPGX, where the number after G represents the volume of PEG in the mixture.

### 2.3. Characterization of the Self-Gelling PPG Powder

We studied the self-gelation time of the self-gelling PPG powder using the inverted tube method.

The chemical structures and their interaction between components were analyzed using Fourier transform infrared spectroscopy (FTIR, Nicolet 6700, Thermo Fisher, Waltham, MA, USA) in the wavenumber region of 4000 cm^−1^ to 500 cm^−1^.

The seat drop method was performed with a contact angle tester (SDC 350KS, Kunshan Shengding, Suzhou, China) to study the contact angle of the self-gelling PPG powder.

The microstructures and surface morphology of self-gelling PPG powder were characterized using scanning electron microscopy (SEM, Nova NanoSEM450, FEI, Hillsboro, OR, USA) under an accelerating voltage of 20 kV after spraying with gold.

A dynamic rheological experiment was conducted to study the viscoelasticity of the self-gelling PPG powder-derived hydrogel using a rheometer (MCR 302, Anton Paar, Graz, Austria) with a 20 mm parallel plate. Briefly, 0.2 g of the self-gelling PPG powder was placed on parallel plates preheated to 37 °C. Next, phosphate-buffered saline (PBS) (0.01 M, pH 7.4) was added dropwise to the self-gelling PPG powder. Then, the powder was evenly immersed with PBS to prepare a self-gelling PPG powder-derived hydrogel. The storage modulus G′ and loss modulus G′′ tests were tested at 37 °C within the frequency range of 0.1–10 Hz and at a strain of 1%.

### 2.4. The Water Absorption Capacity of the Self-Gelling PPG Powder

The water absorption capacity of the self-gelling powders with different compositions was tested by immersing them in PBS at room temperature until they reached equilibrium. Briefly, the lyophilized sample was weighed as *W_0_* and was immersed in 25 mL of PBS for a specific time (1 min, 2 min, 3 min, 5 min, 8 min, 10 min, 15 min, 20 min, 30 min, 50 min, 80 min, 120 min, and 150 min). After a set period of soaking, the sample was removed from the solution, and the surface water of these samples was dried with filter papers and weighed as *W*_1_. The swelling ratio of the self-gelling PPG powders was calculated according to Equation (1).

(1) $$\mathrm{Water}\;\mathrm{uptake}\;\mathrm{ratio}\;\left(\%\right)=\frac{W_{1}-W_{0}}{W_{0}}\times 100$$

### 2.5. Tissue Adhesion Experiments

The adhesion performance of the self-gelling PPG powder on porcine skin was tested in lap shear experiments using a universal electromechanical tester (WDW-05, Si Pai Inc., Shanghai, China). For the adhesion test, fresh porcine skin (50 × 20 mm) was polished and washed with DI water and ethanol before being utilized. Then, the self-gelling PPG powders with different ratios were placed evenly over a 20 × 20 mm square area (*S*) on the skin tissue and hydrated with a certain amount of PBS on the self-gelling PPG powders. Subsequently, another piece of porcine skin was quickly pressed onto the powder-coated porcine skin and pinned down at room temperature for 60 min; then, it was peeled at 180° with a fixed pulling speed of 10 mm/min on a universal electromechanical tester until the hydrogel was completely peeled off of the porcine skin, and the maximum peeling force (*F_max_*) exerted during the stretching process was recorded. The adhesion stress was calculated using Equation (2).

(2) $$\mathrm{Adhesion}\;\mathrm{stress}=\frac{F_{max}}{S}$$

### 2.6. In Vitro Hemolysis Assay

An in vitro hemolysis assay was conducted using freshly collected whole blood samples from rabbits to measure the biocompatibility of the self-gelling PPG powder. First, the whole blood samples from rabbits were centrifuged at 3000 rpm for 25 min at room temperature and washed three times with a certain amount of PBS solution to collect precipitated red blood cells (RBCs). Then, the obtained RBCs were diluted in an adequate volume of PBS to obtain a 2% erythrocyte suspension. Subsequently, 50 mg of the self-gelling PPG powders with different compositions were selected as the experimental groups and were added to the prepared RBC suspension; then, they were incubated at 37 °C for 2 h. In addition, PBS and deionized water were set as negative and positive controls. These mixtures were incubated under the same conditions. Finally, all samples were centrifuged for 10 min to obtain a supernatant. The absorbance of the supernatant was investigated three times with a microplate reader at 540 nm. The hemolysis ratio of all samples was calculated using Equation (3).

(3) $$\mathrm{Hemolysis}\;\mathrm{ratio}\;(\%)=\frac{{OD}_{sample}-{OD}_{negative}}{{OD}_{positive}-{OD}_{negative}}\;\times \;100$$

where *OD_sample_*, *OD_negative_*, and *OD_positive_* are the absorbance values of the sample, negative control (PBS), and positive control (DI water), respectively.

### 2.7. Antibacterial Activity Test

The antibacterial abilities of the self-gelling PPG powders were evaluated using the inhibition rate against Escherichia coli (*E. coli*, CMCC44102, Gram-negative) and Staphylococcus aureus (*S. aureus*, CMCC26003, Gram-positive). The coated plate method was used to measure the antimicrobial properties of the self-gelling PPG powders. Firstly, self-gelling PPG powder and 10 wt% solution of PEI were added to bacterial stock suspensions of 1 mL of *E. coli* and 1 mL of *S. aureus* (1 × 10^7^ CFU/mL), respectively. The same volumes of bacterial stock suspensions of E. coli and S. aureus without adding any substances were used as the positive control groups. Then, they were incubated at 37 °C for 12 h. The resulting bacterial stock suspensions were evenly plated on lysogeny broth (LB) medium after being diluted to a concentration of 10^4^ CFU/mL, and then the bacteria and their colonies that formed on each LB medium were photographed and counted after incubation at 37 °C for 12 h in a bacterial incubator. For each group, the test was repeated three times, and the ratio was calculated using Equation (4).

(4) $$\mathrm{Antibacterial}\;\mathrm{rate}\;(\%)=\frac{A_{B}-A_{D}}{A_{B}}\times 100\;$$

where *A_B_* is the number of surviving bacteria in the blank control group, and *A_D_* is the number of surviving bacteria in the determined sample group.

### 2.8. In Vivo Hemostatic Assay

The in vivo hemostatic effect was measured using four models: a liver bleeding model, a tail bleeding model, and ear vein and artery bleeding models. All animal experiments were executed according to the Guidelines for Care and Use of Laboratory Animals of Hubei University of Science and Technology and approved by the Animal Ethics Committee of Hubei University of Science and Technology, and all animal procedures were conducted in terms of the Guidelines for the Care and Use of Laboratory Animal of Hubei University of Science and Technology. For the rat liver and tail vein bleeding models, 15 SD rats (8 weeks, female, 180–200 g, Beijing HFK Bio-Technology Co., Ltd., Beijing, China) were randomly divided into 3 groups: control, gauze, and PPG4. In the rat liver hemostasis model, SD rats were anesthetized with 2% pentobarbital and fixed on a surgical operating table. Subsequently, the left lobe of the rat liver was exposed through a longitudinal abdominal incision and then punctured with a 22 G needle. Next, gauze and self-gelling powder were immediately applied to the bleeding areas, and the duration of the bleeding process was recorded using a digital camera at certain times. At the same time, the untreated bleeding liver model was used as a control. After the bleeding stopped, the amount of blood loss and the hemostatic time of the different treatment methods were evaluated (n = 5). For the bleeding model involving rat tail amputation, the part about 2–3 cm away from the tip of the rat’s tail was cut off, and then the gauze and PPG4 powder were used on the wound surface while the untreated wound was set as the control. The hemostatic process was similar to that of the liver bleeding model. The amount of blood lost and the hemostatic time were recorded (n = 5). For the ear vein and artery bleeding models in rabbits, the experiments were conducted on male rabbits (1.8–2 kg, Beijing HFK Bio-Technology Co., Ltd., Beijing, China). All rabbits were anesthetized with 2 wt% pentobarbital and fixed on the surgical operating table. Subsequently, the ear vein and artery of the rabbits were punctured by using a 22 G medical syringe needle to induce bleeding, and then the PPG4 self-gelling powders were put on the bleeding site for treatment (n = 5).

### 2.9. Statistical Analysis

Each experimental datum is expressed as the mean ± standard deviation (SD) from at least three independent experiments (n ≥ 3). Significant differences were assessed using one-way ANOVA. Statistical significance was treated as *** *p* < 0.001, ** *p* < 0.01, and * *p* < 0.05, while ns means no significant difference.

## 3. Results and Discussion

### 3.1. Gelation Time of the Self-Gelling PPG Powder

**Figure 2:**
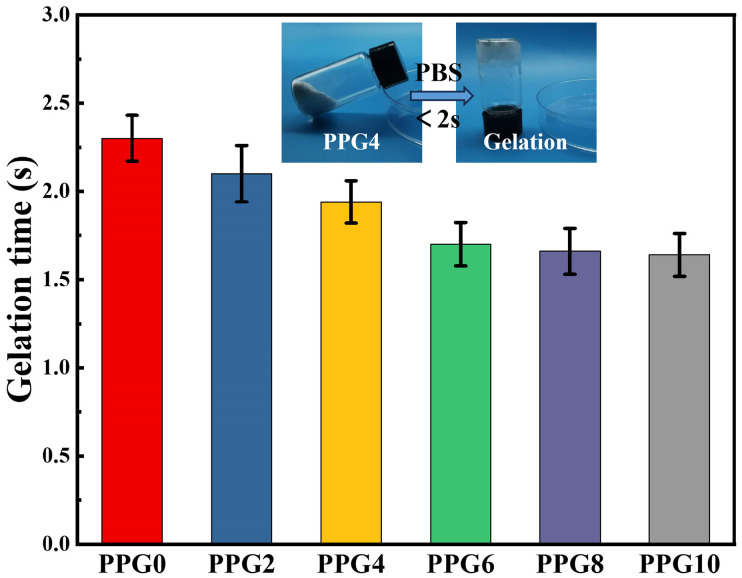
Gelation time of the self-gelling PPG powder.

PPG powders were prepared through lyophilization and by grinding their complexes. Meanwhile, they can absorb liquid quickly and form hydrogels (Figure 2). In addition, with increasing mass ratios of PEG, the PPG powder showed a shortened self-gelation time from 2.30 ± 0.13 s to 1.64 ± 0.12 s (Figure 2). The possible reason for this phenomenon was that the presence of the hydrophilic PEG component clearly enhanced the surface wettability and liquid absorption capacity of the self-gelling PPG powder, which will be confirmed by the contact angle data later.

### 3.2. FTIR Analysis

**Figure 3:**
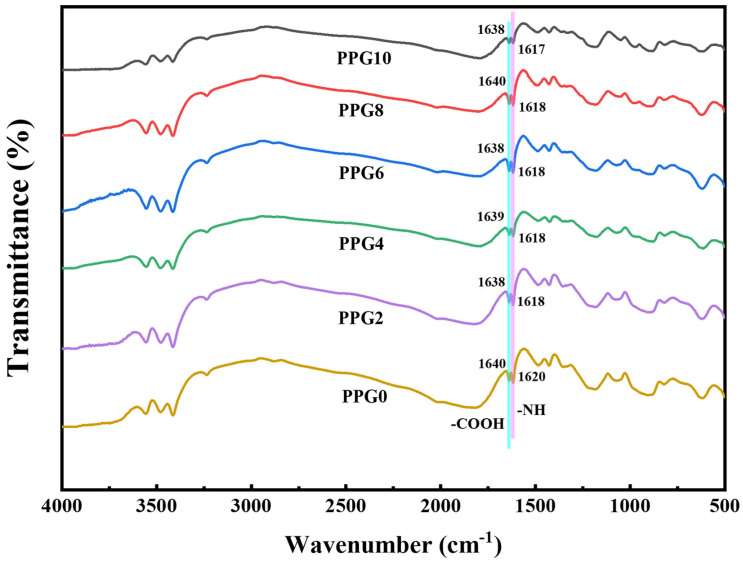
FTIR spectra of the self-gelling PPG powder.

FTIR spectroscopy was performed to analyze the chemical structures and their interactions in the self-gelling PPG powders with different compositions. As shown in Figure 3, which compares the FTIR spectra of the self-gelling PPG powders with different compositions, there were no significant differences, and the slight shifting in the characteristic carboxylic acid (-COOH) and amine group peaks (-NH or -NH_2_) in the self-gelling PPG powders with different compositions without generating new characteristic peaks showed that PEI, PAA, and PEG were crosslinked through physical interactions, such as hydrogen bonding and electrostatic interaction, instead of covalent bonds [24].

### 3.3. Contact Angle (θ_H2O_) of the Self-Gelling PPG Powder

**Figure 4:**
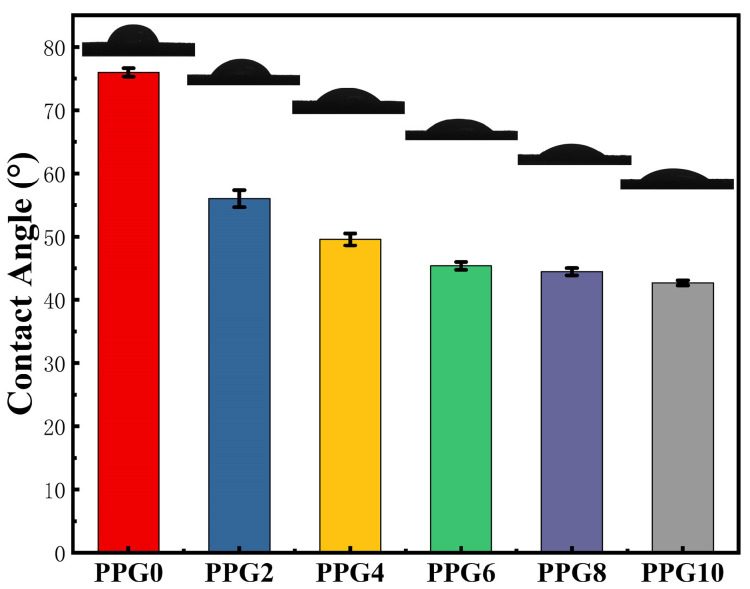
Water contact angles of the self-gelling PPG powder.

The water contact angle (*θ_H2O_*) is commonly used to evaluate the hydrophilicity and hydrophobicity of materials. The water contact angle of the self-gelling PPG powders with different compositions was investigated to characterize their interfacial hydrophilicity and showed a trend toward lower angles at a higher PEG content. As shown in Figure 4, the water contact angles of the self-gelling PPG0, PPG2, PPG4, PPG6, PPG8, and PPG10 powders were 75.94 ± 0.67°, 56.00 ± 1.34°, 49.56 ± 0.95°, 45.38 ± 0.61°, 44.46 ± 0.55°, and 42.66 ± 0.40°, respectively, implying that the presence of hydrophilic PEG content significantly enhanced the surface wettability of the PPG powders.

### 3.4. SEM Analysis

**Figure 5:**
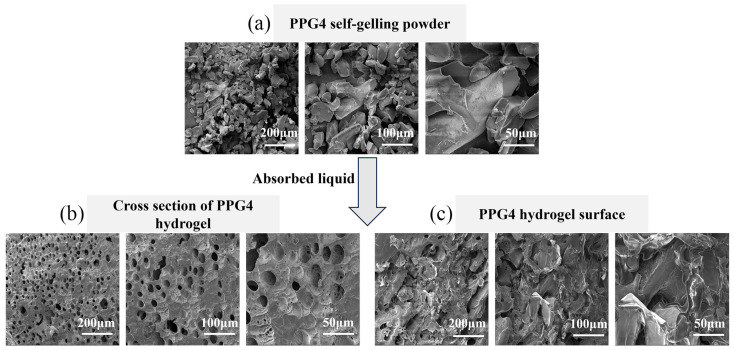
(**a**) SEM images of the self-gelling PPG powder. (**b**) Cross-section images of the self-gelling PPG powder-derived hydrogel after absorbing liquid. (**c**) SEM images of the self-gelling PPG powder-derived hydrogel surface after absorbing liquid.

The microstructures and surface morphology of the self-gelling PPG powders were characterized using SEM. When the self-gelling PPG powder particles (Figure 5a) became a PPG powder-derived hydrogel after absorbing the liquid, the self-gel exhibited a three-dimensional porous structure (Figure 5b), and there were no significant cracks on the surface of the formed gels (Figure 5c). The three-dimensional porous structure could rapidly absorb body liquid exuded from damaged tissue, thereby promoting hemostasis.

### 3.5. Rheological Analysis

**Figure 6:**
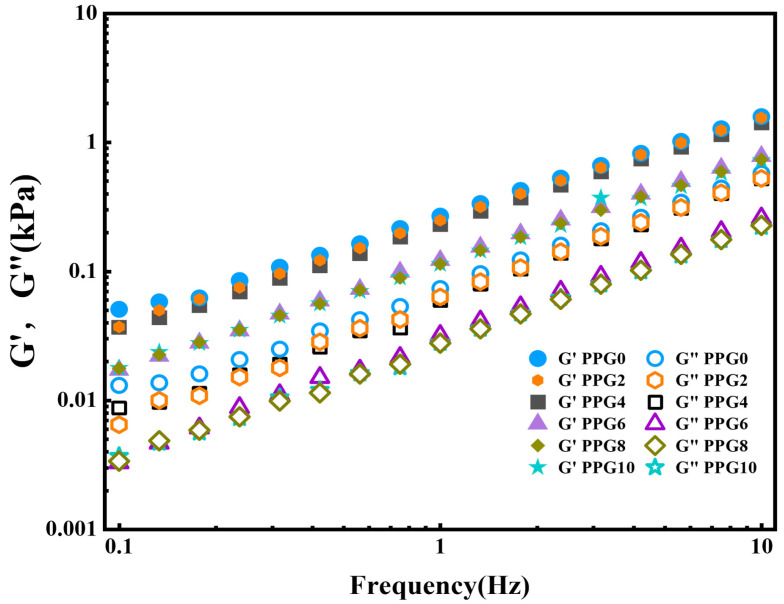
The dynamic rheological behaviors of the hydrogel formed by the self-gelling PPG powder.

The rheological characteristics of the hydrogel formed by the self-gelling PPG powder were analyzed to measure its viscoelastic behavior. As shown in Figure 6, for all samples, the storage modulus (G′) was consistently higher than the loss modulus (G″), indicating that the self-gelling PPG powder could form a gel in a stable elastic state [25,26].

### 3.6. Water Absorption Capacity of the Self-Gelling PPG Powder

**Figure 7:**
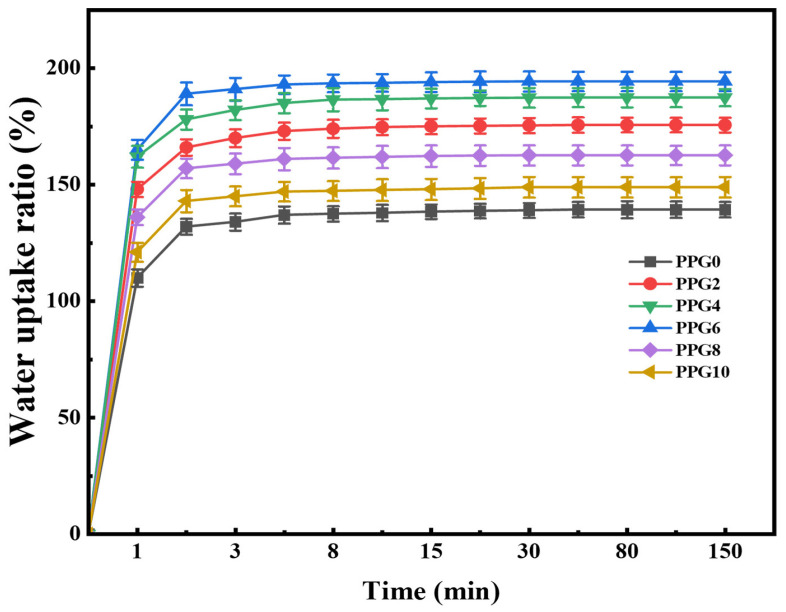
Water absorption capacity of self-gelling PPG powder.

The water absorption capacity of hemostatic powders plays an important role in increasing the concentration of coagulation factors and activating the cascade reaction in bleeding tissue [12]. Thus, we performed a swelling experiment to investigate the absorption effect of the self-gelling PPG powders with different compositions. As shown in Figure 7, all powders rapidly absorbed lots of water, eventually reaching an equilibrium state within 60 min, and the content of PEG was the main factor regulating the water absorption capacity of the self-gelling PPG powders with different compositions. In detail, the PPG0 powder showed a limited water absorption capacity with a maximum value of 139.3%± 3.31%. Meanwhile, as the content of PEG increased, PPG2 powder, PPG4 powder, and PPG6 powder showed a growing water absorption ratio of 175.6% ± 3.16%, 187.4 ± 3.71%, and 194.3 ± 4.11%, respectively. It has been reported that the presence of a hydrophilic PEG component significantly enhances the surface wettability and water absorption capacity of materials [27], which is in agreement with the above results. However, it was worth noting that the self-gelling PPG8 and PPG10 powders, despite having the highest content of PEG, did not exhibit the highest swelling ratios; the reason might be that the introduction of excess PEG disrupts the electrostatic cross-linking network structure between PEI and PAA, further restricting the absorption and retention of water molecules during the swelling process and resulting in reduced water absorption capacity.

### 3.7. Adhesion Performance Test of the Self-Gelling PPG Powder-Derived Hydrogel

**Figure 8:**
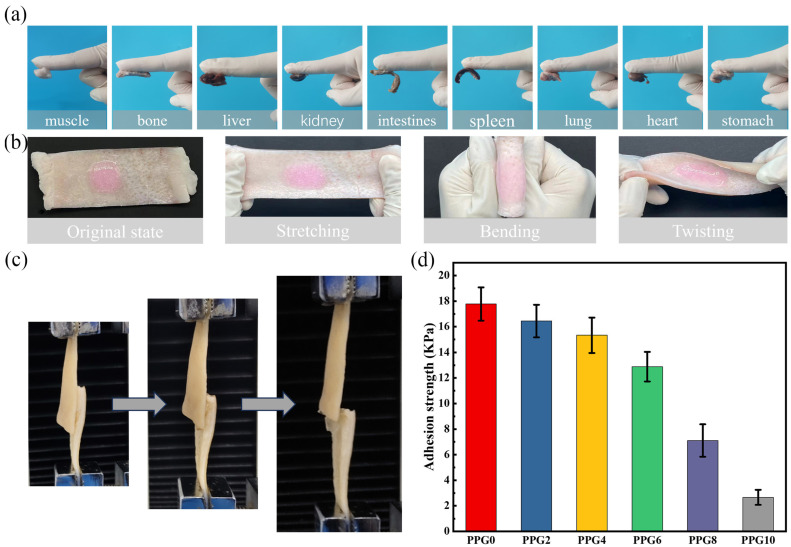
Tissue adhesive properties of the self-gelling PPG powder-derived hydrogel: (**a**) adhesion of the self-gelling PPG4 powder-derived hydrogel on various tissue surfaces, including muscle, bone, liver, kidney, intestines, spleen, lung, heart, and stomach. (**b**) The self-gelling PPG4 powder-derived hydrogel strongly adhered to porcine skin tissues under various actions (original state, stretching, bending, and twisting; the hydrogel was dyed with rhodamine B). (**c**) Lap shear test steps of hydrogels. (**d**) Adhesion strength of the self-gelling PPG powder-derived hydrogel.

The capacity for adhesion with bleeding tissue is the main parameter for enhancing the hemostatic effect of a powder. The adhesion abilities were tested using the tissue attachment method and lap shear test, as described above. After contact with water, the PPG powders could form a stable hydrogel and adhere to the tissue surface. As shown in Figure 8a, the self-gelling PPG4 powder-derived hydrogel could steadily adhere to different tissue surfaces, including the muscle, bone, liver, kidney, intestines, spleen, lung, heart, and stomach, and Figure 8b shows that when the self-gelling PPG4 powder-derived hydrogel adhered to porcine skin in situ, it could firmly accommodate complex deformations, such as stretching, bending, and twisting (the hydrogel was dyed with rhodamine B for better visualization). The above results indicate that the self-gelling PPG powders could form gels with robust adhesion to the surface of biological tissues. The adhesion performance was also measured using a lap shear test (Figure 8c). As shown in Figure 8d, as the content of PEG increased, the adhesion strength of the self-gelling PPG powder-derived hydrogel decreased from 17.7 ± 1.3 kPa to 2.6 ± 0.59 kPa. One possible explanation for this phenomenon is that introducing PEG attenuates the electrostatic interaction between PEI and PAA. Although the adhesion strength of self-gelling PPG powder-derived hydrogel decreased with the increase in the content of PEG, the self-gelling PPG0, PPG2, PPG4, and PPG6 powder-derived hydrogels still had better adhesion performance than that of other hemostatic materials [12,28].

### 3.8. In Vitro Hemolysis Assay

**Figure 9:**
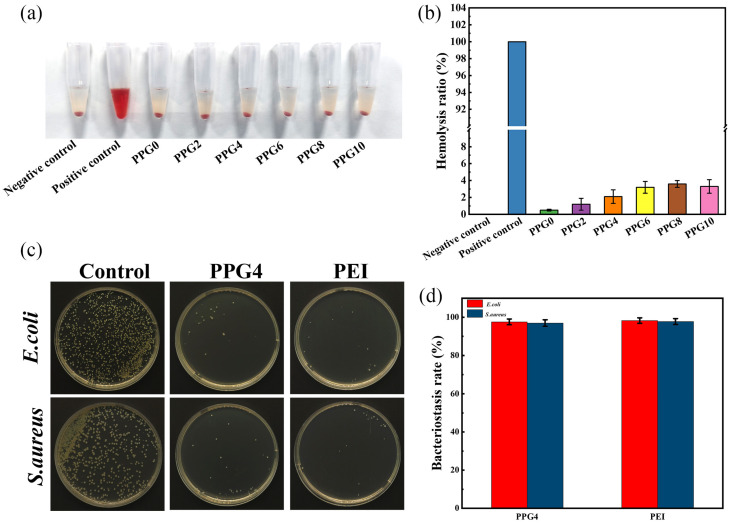
In vitro hemolysis assay and antibacterial performance. (**a**) Digital images of the hemocompatibility of self-gelling PPG powder with different compositions. (**b**) The hemolysis ratios of self-gelling PPG powder with different compositions. (**c**) Bacteriostatic effects of different samples on *S. aureus* and *E. coli*. (**d**) Bacterial inhibition rates of different samples against *S. aureus* and *E. coli*.

Biosafety is a crucial element of materials applied in the biomedical field. To evaluate the hemocompatibility of self-gelling PPG powders with different compositions, an in vitro hemolysis test was performed. The hemolysis test was conducted using DI water as a positive control and PBS as a negative control., as shown in Figure 9a. The results showed that the hemolysis rates of self-gelling PPG powders with different compositions were all below 5% (Figure 9b), which indicated that all self-gelling PPG powders had excellent hematological compatibility.

### 3.9. Antibacterial Capabilities of Self-Gelling PPG Powder

To balance the hydrophilic properties, tissue adhesion, and biocompatibility of the material, the self-gelling PPG4 powder was selected for subsequent experiments.

The antibacterial ability of the self-gelling PPG4 powder against *S. aureus* and *E. coli* was assessed using the coated plate method. Bacterial suspensions were incubated with the self-gelling PPG4 powder and 10 wt% solution of PEI at 37 °C for 12 h, respectively. As shown in Figure 9c,d, the results showed that, compared with the control group, the self-gelling PPG4 powder groups exhibited an excellent bacteriostasis rate against *S. aureus* (97% ± 1.7%) and *E. coli* (97.6% ± 1.5%), and the bactericidal ratio was similar to that of the 10 wt% solution of PEI (97.8% ± 1.54% and 98.3% ± 1.42% against *S. aureus* and *E. coli*, respectively). These results show that the antibacterial activity is mainly because cationic amine groups of polyethyleneimine (PEI) are able to capture and kill bacteria with a negatively charged cell membrane on contact, as shown in the previous reports [29].

### 3.10. In Vivo Hemostatic Assay

**Figure 10:**
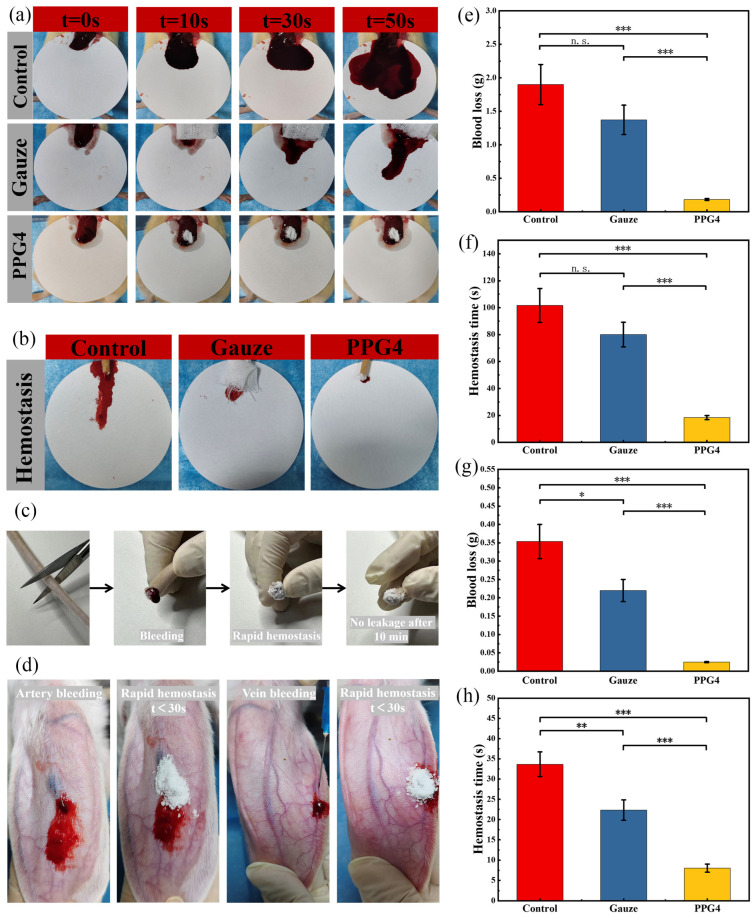
In vivo hemostatic performance of different materials. (**a**) Photos of the hemostatic effects of different hemostatic materials on the rat liver bleeding model. (**b**) Photos of the hemostatic effects of different hemostatic materials on the rat tail amputation bleeding model. (**c**) Photos of the hemostatic effect of self-gelling PPG4 powder on the rat tail amputation bleeding model. (**d**) Photos of hemostatic effects of self-gelling PPG4 powder in the ear vein and artery bleeding experiments in rabbits. (**e**) The amount of bleeding and (**f**) hemostatic time in the rat liver blood loss model. (**g**) The amount of bleeding and (**h**) hemostatic time in the rat tail amputation bleeding model (n.s.: no significant difference, * *p* < 0.05, ** *p* < 0.01, *** *p* < 0.001).

The hemostatic ability of different hemostatic materials in vivo was measured using a liver bleeding model, tail bleeding model, and ear vein and artery bleeding models. In the rat liver hemorrhage model (Figure 10a), as shown in Figure 10e,f, the self-gelling PPG4 powder significantly decreased the blood loss to 183.4 ± 13.5 mg, and the bleeding time was shortened to 18.3 ± 1.52 s; the hemostatic effect was clearly better than that in the control group (1900.0 ± 310 mg, 101.6 ± 12.5 s) (*** *p* < 0.001) and gauze group (1373 ± 218.8 mg, 80 ± 9.1 s) (*** *p* < 0.001) because the self-gelling PPG4 powder was able to absorb a significant amount of blood on the bleeding tissue surface and form a physical hemostatic barrier, but gauze could not. In addition, the hydrophilic PEG component was able to clearly improve the interfacial liquid uptake and surface wettability of the self-gelling PPG powder, as shown in Figure 2 and Figure 4; this was the main parameter for regulating the hemostatic performance and quickly swelling into a hemostatic micro-gel network of self-gelling PPG powder.

The self-gelling PPG4 powder also showed excellent hemostatic activity for the bleeding model of rat tail amputation (Figure 10b,c,g,h). As shown in Figure 10g,h, the self-gelling PPG4 powder decreased the blood loss to 24.6.4 ± 1.5 mg, and the bleeding time was shortened to 8.3 ± 1.12 s; the hemostatic effect was clearly better than that in the control group (353.3 ± 46.5 mg, 33.6 ± 3.05 s) (*** *p* < 0.001)and gauze group (220.0 ± 30.0 mg, 22.3 ± 2.52 s) (*** *p* < 0.001).

We further demonstrated the capacity of self-gelling PPG4 powders to control bleeding in terms of the ear vein and artery bleeding experiments in rabbits. After placing the self-gelling PPG4 powders on the damaged tissue surface, the powders could form a physical hemostatic barrier by absorbing interfacial blood and quickly controlled bleeding (ear vein bleeding <20 s, ear artery bleeding <30 s), as shown in Figure 10d.

## 4. Conclusions

In summary, we successfully synthesized a self-gelling powder via a simple preparation method that can quickly absorb blood and tissue fluid and rapidly form a stable, robust hydrogel as a physical barrier to achieve effective hemostasis even for irregularly shaped and non-compressible wounds. The high adhesion of this self-gelling PPG powder-derived hydrogel allows it to be applied to wounds with broken and bleeding blood vessels to resist the pressure of strong blood flow. Furthermore, the biosafety was evaluated in an in vitro hemolysis test. In addition, the self-gelling powder PPG can inhibit bacteria, showing inhibition of both Gram-negative and Gram-positive bacteria. Moreover, the effect of the powder on promoting hemostasis was confirmed through a rat liver bleeding model, a rat tail bleeding model, and rabbit ear vein and artery bleeding models, demonstrating its ability to effectively stop bleeding. Due to its rapid and effective hemostatic performance, adaptability for satisfying various complex bleeding wounds and non-compressible sites, excellent antimicrobial properties and hemocompatibility, easy usage, and low cost, we believe that self-gelling PPG4 powder is a promising biomedical material with wide application prospects in the field of medicine.

## Figures and Tables

**Table 1:** Compositions of the self-gelling PPG powder.

Sample	10 wt% PEI (mL)	10 wt% PAA (mL)	10 wt% PEG (mL)
PPG0	10	10	0
PPG2	10	10	2
PPG4	10	10	4
PPG6	10	10	6
PPG8	10	10	8
PPG10	10	10	10

## Data Availability

The original contributions presented in this study are included in the article, and further inquiries can be directed to the corresponding author.
